# Vaccination of mice with *Trichinella spiralis* serine proteinase enhanced gut epithelial barrier and elicited a high protective immunity

**DOI:** 10.1371/journal.pntd.0014161

**Published:** 2026-03-24

**Authors:** Jin Yi Wu, Xin Zhuo Zhang, Ru Zhang, Yao Zhang, Ruo Dan Liu, Xi Zhang, Shao Rong Long, Zhong Quan Wang, Jing Cui

**Affiliations:** Department of Parasitology, School of Basic Medical Sciences, Zhengzhou University, Zhengzhou, China; Xuzhou Medical University, CHINA

## Abstract

**Background:**

A *Trichinella spiralis* serine proteinase (TsSPc) was identified in the intestinal infective larva (IIL) surface and excretory-secretory (ES) antigens. Our previous study showed that recombinant TsSPc (rTsSPc) disrupted intestinal epithelial integrity and barrier function, and mediated larval invasion of intestinal mucosa. This study aims to investigate the impact of rTsSPc vaccination on intestinal epithelial integrity and its elicited protective immunity in a mouse model.

**Methodology/principal finding:**

ELISA results demonstrated that rTsSPc immunization induced a systemic humoral immune response with serum-specific IgG antibody titer reaching 1:10⁵, and elicited a mixed Th1/Th2 immune response dominated by the Th2 type. In the rTsSPc-immunized mice, the TsSPc-specific intestinal sIgA level was also markedly increased (*P* < 0.0001); The secretion levels of IFN-γ and IL-4 in spleen, mesenteric lymph node (MLN) and Peyer’s patch (PP) cells were significantly increased (*P* < 0.0001). rTsSPc immunization blocked the binding of parasite-derived TsSPc and gut epithelial RACK 1 receptor, prevented the activation of MAPK/ERK1/2 pathway and enhanced gut epithelial integrity, and impeded the parasite invasion. Vaccination of mice with rTsSPc exhibited a 65.7% reduction of enteral adult burden with a 61.13% decline of female fecundity, and a 58.10% reduction of muscle larval burden. Intestinal inflammation of rTsSPc-immunized mice was also significantly alleviated, as demonstrated that goblet cell numbers were obviously decreased, expression level of mucins (Muc2 and Muc5ac) and pro-inflammatory cytokines (IL-6 and TNF-α) was evidently declined, while expression level of anti-inflammatory cytokines (IL-10 and TGF-β) was distinctly increased after infection. Moreover, peritoneal macrophages of rTsSPc-immunized mice exhibited a mixed M1/M2 polarization, but shifted to a predominant M2 polarization pattern post infection. ADCC assay confirmed that peritoneal macrophage of immunized mice had a stronger anti-rTsSPc antibodies-mediated cytotoxicity killing newborn larvae (*P* < 0.0001).

**Conclusions:**

rTsSPc vaccination produced a high protective immunity through multiple synergistic mechanisms: eliciting an obvious humoral and cellular immunity, gut local mucosal sIgA responses; blocking the binding of parasite-derived TsSPc to gut RACK1 receptors and the activation of MAPK/ERK1/2 pathway, improved gut epithelial integrity, inhibiting larval invasion, enhancing macrophages’ ability of ADCC killing larvae, and finally reduced parasite burden and alleviated inflammation of intestines and skeletal muscles. TsSPc might be a promising novel candidate target for anti-*T. spiralis* vaccine.

## Author summary

*T. spiralis* serine proteinase (TsSPc) plays a crucial role in larval invasion of host intestinal epithelium and disruption of intestinal epithelial integrity. Previous studies demonstrated that rTsSPc specifically bound to gut epithelial RACK 1 receptor and activated MAPK/ERK1/2 pathway, decreased the tight junctions (TJs) expression and damaged gut barrier function, and mediated larval intrusion of gut mucosa. The purpose of this study was to investigate the impact of rTsSPc vaccination on intestinal epithelial integrity and its triggered protective immunity. The results showed that subcutaneous vaccination of mice with rTsSPc triggered a strong systemic humoral immune response (high levels of serum-specific IgG, IgG1/IgG2a, IgA) and gut mucosal sIgA responses. The levels of Th1/Th2 cytokines (IFN-γ/IL-4) secreted by spleen, mesenteric lymph nodes, and Peyer’s patches were significantly increased at 6 weeks post-vaccination. rTsSPc immunization blocked the binding of parasite-derived TsSPc to gut epithelial RACK1 receptor and activation of MAPK/ERK1/2 pathway, enhanced gut epithelial integrity, and impeded the parasite invasion. The rTsSPc-vaccinated mice exhibited a 65.7% and 58.1% reduction of gut adult burden and muscle larval burdens, respectively. Moreover, the numbers of goblet cells and expression levels of Muc2, Muc5ac and pro-inflammatory cytokines (TNF-α and IL-6) in immunized mice were notably decreased after infection, while the expression of anti-inflammatory cytokines (IL-10 and TGF-β) was obviously elevated. Additionally, peritoneal macrophages of rTsSPc-immunized mice exhibited a mixed M1/M2 polarization, but shifted to a predominant M2 polarization pattern post infection. ADCC assay confirmed that peritoneal macrophage of immunized mice had a stronger anti-rTsSPc antibodies-mediated cytotoxicity killing newborn larvae. TsSPc might be a promising novel candidate target for anti-*T. spiralis* vaccine.

## Introduction

Trichinellosis is a globally distributed zoonotic parasitic disease caused by the nematode of genus *Trichinella*, and has a significant threat to global public health and pork food safety. Trichinellosis is acquired by the consumption of raw or undercooked meat contaminated with the muscle larvae (ML), with pork being the main source of human infection in most countries [1]. From 2009 to 2020, a total of eight human trichinellosis outbreaks involving 479 cases and two deaths were reported in China, and seven (87.50%) out of eight outbreaks were attributed to the consumption of raw or semi-cooked pork [2]. Only in 2023, 76 confirmed human cases were reported in 11 member states of the European Union [3]. *T. spiralis* infection not only causes swine growth retardation and reproductive disorders, but also results in substantial economic losses due to pork inspection failures [4]. Currently, the prevention and control of *Trichinella* infection mainly depend on meat safety inspections and anthelmintic drugs, but there are no approved prophylactic vaccine available [5]. Therefore, the development of preventive anti-*Trichinella* vaccine is urgently needed to control this nematode infection in domestic food animals [6].

The life cycle of *T. spiralis* consists of two different developmental stages: intestinal stage and muscle stage. After the encapsulated ML in meat are digested by gastric juices, the ML are released from their collagen capsules, and the larvae are activated into intestinal infective larvae (IIL), which invade intestinal epithelial cells (IECs), undergo four molts and develop into adult worms (AW) [7]. Subsequently, female and mate AWs mate, and pregnant females produce the newborn larvae (NBL), the NBL migrate to skeletal muscles through the blood circulatory system and develop into the encapsulated ML to complete the life cycle [8]. Among the various worm stages, the IIL stage is the most critical worm stage to invade gut mucosa and establishes infection, and the invasive process is principally mediated by the invasive molecules and proteases in surface proteins/excretory-secretory (ES) proteins secreted by the IIL stage [9,10]. Among these ES proteins and proteases, serine proteases constitute a large and conserved family characterized by a catalytic serine residue in their active center, and play multiple critical roles in parasite pathogenicity [11]. Serine proteases hydrolyze host extracellular matrix (ECM) and gut epithelial intercellular tight junction (TJ) proteins to facilitate larval penetration, regulate host immune responses to achieve the immune evasion, and participate in larval molting and female reproduction [12,13]. Therefore, serine proteases have been regarded as potential candidate molecules of vaccines against *Trichinella* invasion and infection [14,15].

In previous studies, a *T. spiralis* serine proteinase (TsSPc, GenBank: U62659.1) was identified in the surface and IIL ES proteins. TsSPc is a secretory serine protease which was expressed throughout all developmental stages of the parasite, and primarily localized in the cuticle and stichosome [16]. Recombinant TsSPc (rTsSPc) specifically bound to intestinal epithelial receptor for activated C kinase 1 (RACK1) and cytokeratin 8 (CK8), activated various pathways (MAPK/ERK1/2 or RhoA/ROCK1), destroyed the TJs proteins and gut epithelial integrity, thereby mediated larval invasion of gut epithelium [17,18]. Additionally, rTsSPc also could specifically bind with PGAM5 receptors in IECs, triggered gut epithelial apoptosis, reduced the TJs expression and damaged gut barrier function, and promoted larval invasion of gut mucosa [19,20], suggesting that TsSPc might be a potential target molecule for developing anti-*Trichinella* vaccines.

The purpose of this study was to assess the impact of rTsSPc vaccination on intestinal epithelial integrity and its elicited protective immunity in a mouse model.

## Materials and methods

### Ethics statement

This study was performed in light of the National Guidelines for Experimental Animal Welfare (Minister of Science and Technology, People’s Republic of China, 2006). All animal experiments in this study were approved by the institutional Life Science Ethics Committee of Zhengzhou University (No. ZZUIRB GZR 2023–1397).

### *Trichinella* species and experimental animal

*Trichinella spiralis* (ISS534) was collected from a naturally infected swine in Henan province of China, and maintained by serial passage in BALB/c mice in our department [21]. The female BALB/c mice with 6–8 weeks old were purchased from the Experimental Animal Center of Zhengzhou University.

### Preparation of rTsSPc protein

The full-length TsSPc cDNA sequence was cloned into pQE-80L to construct recombinant expression plasmid pQE-80L/TsSPc [16]. The pQE-80L/TsSPc was expressed in *E. coli* BL21 (DE3) (Novagen, USA) under induction of 0.5 mM isopropyl β-D-1-thiogalactopyranoside (IPTG) for 6 h at 37 °C, and purified by a Ni-NTA-Sefinose resin containing His tag (Sangon Biotech, China). Finally, rTsSPc was refolded by dialysis and renatured methods [22].

### Subcutaneous immunization of mice with rTsSPc and sample collection

A total of 150 mice were randomly divided into three groups (50 animals per group). The immunization group was subcutaneously vaccinated with 20 µg rTsSPc protein emulsified in ISA 201 adjuvant (SEPPIC, France). Two boosting injections were administered at 2-week intervals using the same dose of rTsSPc combined with ISA 201 adjuvant. Two control groups received either PBS or ISA 201 adjuvant alone. At two weeks following the final vaccination, all mice were orally challenged with 300 *T. spiralis* ML. Serum samples were collected from ten mice per group at weeks 0, 2, 4, 6, 7, 9 and 11 after vaccination. Tail blood (100 µl) was obtained and centrifuged to isolate sera, which were stored at −80 °C until use. To assess intestinal mucosal immune responses, five mice per group were euthanized, intestinal tissues, spleens, mesenteric lymph nodes (MLNs) and Peyer’s patches (PPs) were collected at weeks 0, 6, 7 and 11 after vaccination, their cells were isolated and cultivated to measure the production of cytokines (IFN-γ and IL-4) [23]

**Fig 1:**
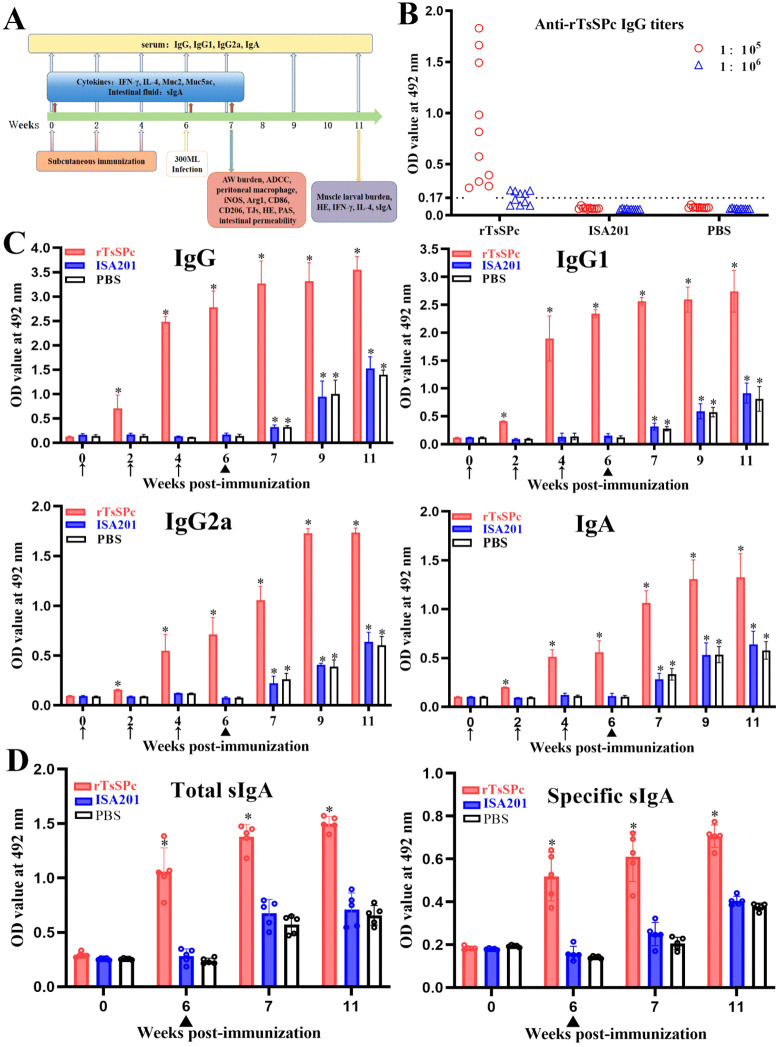
The designed vaccination scheme and anti-rTsSPc antibody detection. **A:** The designed vaccination scheme and detection protocol. Mice were subcutaneously vaccinated with 20 µg rTsSPc protein emulsified in ISA 201 adjuvant three times at weeks 0, 2, and 4. At two weeks following the final vaccination, the vaccinated mice were orally challenged with 300 *T. spiralis* ML. Anti-rTsSPc antibodies (IgG, IgG1/IgG2a, and IgA) were measured by rTsSPc-ELISA at weeks 0, 2, 4, and 6 post-immunization, respectively, and at weeks 7, 9 and 11 post-immunization (e.g., 1, 3, and 5 weeks after infection). Five mice per group were euthanized at 6 weeks after immunization, 1 and 5 weeks after infection to assess levels of sIgA and cytokines (IFN-γ and IL-4). At 1 and 5 weeks after infection, ten mice per group were sacrificed to determine intestinal adult burden, female fecundity, and muscle larval burden (larvae per gram of skeletal muscles, LPG). Histopathological examination of intestines and muscles from infected mice was performed. Furthermore, peritoneal macrophages were isolated from ten mice per group at 2 weeks after final immunization and 1 week after infection to evaluate the macrophage polarization. **B:** Serum anti-rTsSPc IgG titers. Anti-rTsSPc IgG levels were assayed in serum sample from vaccinated mice two weeks after the final vaccination. All serum samples were tested in duplicate. The data represent the OD values of anti-rTsSPc IgG levels derived from 10 vaccinated mice. Additionally, 45 serum samples from normal mice diluted at 1:100 were measured as negative controls. The ELISA cut-off values were calculated based on 2.1-fold of the mean OD value of negative control sera from normal mice. Serum samples with OD values equal to or greater than the cut-off were defined as positive. The cut-off value (0.17) is indicated by a dotted line. **C:** Serum anti-rTsSPc antibody kinetics at various times after immunization. The figure illustrates the total IgG response at various time points post vaccination, as well as the specific IgG1/IgG2a subclass responses and IgA levels in vaccinated mice. All serum samples were tested in duplicate, and the OD values are presented as mean ± standard deviation (SD) (n = 10). **D:** Total intestinal sIgA and rTsSPc-specific sIgA levels in enteral washing fluid of vaccinated mice. All enteral fluid samples were assayed in duplicate. No significant sIgA response was detected in the ISA201 or PBS group. The data are presented as mean OD values ± SD (n = 5). Arrows (↑) indicate vaccination times, triangles (▲) denote challenge time. *P < 0.05 compared to the PBS group.

To evaluate the protective efficacy of rTsSPc vaccination, an additional ten mice of each group were sacrificed respectively at 7 and 11 weeks after vaccination, i.e., 1 and 5 weeks after infection [7 days post-infection (dpi) and 35 dpi]. Adult worm burden, female reproductive capacity (the *in vitro* production of NBL deposited by each female for 72 h), and ML burden were assessed as previously described [13]. Furthermore, to investigate intestinal barrier integrity and inflammation reaction, intestinal permeability, goblet cells and expression levels of gut epithelial TJs and mucins, additional five unvaccinated and uninfected mice were also used [19,24]. Moreover, to evaluate the macrophage polarization after rTsSPc immunization and infection, peritoneal macrophages were isolated from ten mice of each group to ascertain macrophage’s surface markers (CD86, CD206), intracellular enzymes (iNOS, Arg1) and cytokine profiles (IL-6, TNF-α, IL-10 and TGF-β). The vaccination schedule is summarized in Fig 1A.

### ELISA determination of serum antibody

Serum specific antibodies (IgG, IgG1/IgG2a and IgA) were measured using an indirect ELISA with rTsSPc as coating antigen [14]. Briefly, 96-well microtiter plates were coated with 100 µl of 1.5 µg/ml rTsSPc and incubated at 4 °C overnight. After washing three times with phosphate-buffered saline containing 0.05% Tween 20 (PBST), the plates were blocked with 5% skim milk in PBST at 37 °C for 2 h. Following being washed again, the plates were incubated at 37 °C for 1 h with primary antibody (immune serum), then with HRP-conjugated goat anti-mouse IgG, IgG1/IgG2a, and IgA (1:10000; Sigma, USA) at 37 °C for 1 h. Finally, the plates were colored using the substrate o-phenylenediamine dihydrochloride (OPD; Sigma) plus 0.15% H_2_O_2_, and reaction was stopped by using 2 M H_2_SO_4_. Optical density (OD) values at 492 nm were measured using a microplate reader [6,25]. Positive results were defined as a ratio of sample OD value to mean OD value of negative serum control ≥ 2.1.

### Sandwich ELISA determining total and specific sIgA in intestinal fluid

The levels of gut secretory IgA (sIgA) in immunized mice were determined using a sandwich ELISA [26]. Briefly, a 20-cm small intestine was cut, and intestinal content was washed three times with 1 ml of cold PBS containing 1% protease inhibitor (Sangon Biotech, Shanghai, China). The washing enteral fluid was collected and centrifuged at 12000 *g* for 5 min at 4 °C, and then supernatants were collected. Total enteral sIgA was measured by a sandwich ELISA, and rTsSPc-specific sIgA was assayed by an indirect ELISA. Color development was performed with OPD substrate, and the OD values at 492 nm were measured using a microplate reader as previously described [26].

### ELISA measurement of cytokines

To ascertain cellular immune response to the rTsSPc immunization, five mice of each group were scarified at 0 and 6 weeks after immunization, and at 1 and 5 weeks after infection. The spleen, MLNs and PPs were recovered from all immunized mice, homogenized in complete DMEM medium (Gibco, Auckland, New Zealand). The cells were collected following centrifugation at 1000 *g* for 5 min, and isolated as reported before [6]. The cell density was adjusted to 2 × 10^6^ cells/ml in DMEM medium containing 5% fetal bovine serum (FBS), 100 U/ml penicillin and 100 μg/ml streptomycin, stimulated with 10 μg/ml rTsSPc at 37˚C and 5% CO_2_ for 72 h [27]. The culture supernatant was obtained and two cytokines (IFN-γ and IL-4) were assayed using a sandwich ELISA (BD Biosciences Pharmingen, USA). The cytokine level was showed as pictograms per milliliter (pg/ml). Additionally, the contents of inflammatory cytokines (TNF-α, IL-6, TGF-β and IL-10) in intestinal fluids were also assayed by ELISA as described before [28].

### Challenge infection and evaluation of immune protection

To evaluate the immune protection induced by rTsSPc vaccination, all immunized mice were orally challenged with 300 *T. spiralis* ML at two weeks after the final immunization. At 7 dpi, AWs were collected from the intestines of ten mice per group. The remaining ten mice from each group were euthanized at 35 dpi. Murine carcasses were weighed, skeletal muscle were artificially digested, and muscle larvae were collected and numbered as previously described [29,30]. The protective efficacy of the rTsSPc vaccination was assessed based on the reduction of intestinal worm burden and larvae per gram (LPG) of skeletal muscles compared to the PBS control group. Additionally, female fecundity was determined as reported before [23,31].

### qPCR and Western blot assay of gut mucosal TJs, mucins and inflammatory cytokines from infected mice

To assess the expression levels of gut mucosal TJs, mucin and inflammatory cytokines of infected mice, mouse intestinal tissues were collected at 7 dpi, qPCR and Western blot assay were performed [19]. Briefly, total RNA was extracted using TRIzol reagent (Takara) and reverse-transcribed into cDNA using a cDNA synthesis kit. Subsequently, qPCR amplification was performed on an ABI Prism 7500 Fast Sequence Detection System (Applied Biosystems, USA). The mRNA levels were normalized to β-actin as an internal reference gene, no significant differences in β-actin expression were observed among diverse groups. PBS negative controls were set for each assay. Relative expression changes of the murine genes were calculated by comparing Ct values using the 2^−ΔΔCt^ method [32]. All experiments were repeated three times. The primers used for qPCR in this study are detailed in Table 1 [33,34]

**Table 1:** Primer sequences of murine TJs, mucin and cytokines for qPCR assays.

Genes	Primers	Sequences (5′end to 3′ end)
Occludin	Forward primer	TGGCAAGCGATCATACCCAGAG
	Reverse primer	CTGCCTGAAGTCATCCACACTC
Claudin-1	Forward primer	GGACTGTGGATGTCCTGCGTTT
	Reverse primer	GCCAATTACCATCAAGGCTCGG
E-cad	Forward primer	AGGACTTCCTGCTGACATCCAG
	Reverse primer	AATCCTGGCAGAACACGGTGCA
Muc2	Forward primer	TGTGGCCTGTGTGGGAACTTT
	Reverse primer	CATAGAGGGCCTGTCCTCAGG
Muc5ac	Forward primer	CTGTGACATTATCCCATAAGCCC
	Reverse primer	AAGGGGTATAGCTGGCCTGA
RACK1	Forward primer	CCCAAGCTTATGACGGAGCATGACC
	Reverse primer	CGGAATTCTTCAGCGGTACCACATGATTA
TNF-α	Forward primer	CCCTCACACTCAGATCATCTTCT
	Reverse primer	GCTACGACGTGGGCTACAG
TGF-β	Forward primer	AGCAACAATTCCTGGCGTTACCT
	Reverse primer	CCTGTATTCCGTCTCCTTGGTTCA
IL-6	Forward primer	TACCACTTCACAAGTCGGAGGC
	Reverse primer	CTGCAAGTGCATCATCGTTGTTC
IL-10	Forward primer	CCCTTTGCTATGGTGTCCTT
	Reverse primer	TGGTTTCTCTTCCCAAGACC
iNOS	Forward primer	GAGAGACAGGGAAGTCTGAAGCAC
	Reverse primer	CCAGCAGTAGTTGCTCCTCTTC
Arg1	Forward primer	CATTGGCTTGCGAGACGTAGAC
	Reverse primer	GCTGAAGGTCTCTTCCATCACC
CD86	Forward primer	CAGACTCCTGTAGACGTGTTC
	Reverse primer	GTCCCATTGAAATAAGCTTGCG
CD206	Forward primer	GTTCACCTGGAGTGATGGTTCTC
	Reverse primer	AGGACATGCCAGGGTCACCTTT
β-actin	Forward primer	CTACCTCATGAAGATCCTGACC
	Reverse primer	CACAGCTTCTCTTTGATGTCAC

For Western blot analysis, mouse enteral tissues collected at 7 dpi were lysed in RIPA buffer and ground on ice for 30 min, followed by centrifugation at 12,000 *g* for 15 min at 4 °C to remove cell debris. Extracted enteral tissue proteins were separated by 10% SDS-PAGE and transferred onto polyvinylidene fluoride membranes (PVDF, Millipore, USA). The membranes were blocked with 5% skim milk in TBST at 37 °C for 2 h, then cut into strips and incubated with primary antibodies against Occludin (1.5 µg/ml), Claudin-1 (1.5 µg/ml), and E-cad (1: 40,000, Abcam, USA), as well as anti-β-actin antibody (1:1000, Servicebio, Wuhan, China) at 4 °C overnight [35,36]. After washing with TBST, the strips were incubated with the secondary antibodies (1:5000 dilutions of HRP-anti-mouse IgG conjugate or HRP-conjugated anti-rabbit IgG; Upingbio, Shenzhen, China) at room temperature for 1 h. Finally, the bands were visualized using an enhanced chemiluminescence kit (ECL; Epizyme, Shanghai, China), and the relative intensity of the bands was analyzed using Image J software (National Institutes of Health, USA) [28].

### Immunofluorescent assay (IFA) and Western blotting of expression of RACK1 and ERK1/2 pathway

To investigate whether rTsSPc immunization prevents the secretion of parasite-derived TsSPc into the mouse intestinal epithelium and blocks its interaction with gut receptor RACK1, small intestines from the rTsSPc, ISA201 and PBS groups were fixed, embedded and sectioned at 7 dpi [16]. The IFA was performed with primary antibodies including anti-rTsSPc serum and anti-RACK1 antibody, followed by secondary antibodies (Alexa Fluor 488 conjugated-goat anti-mouse IgG and Cy3 conjugated-goat anti-rabbit IgG). Nuclei were counterstained red with DAPI [37]. Moreover, mRNA and protein expression of RACK1 and ERK1/2 pathway was also ascertained by qPCR and Western blot as reported before [17].

### Histopathological examination of small intestine and masseter muscles

At 7 and 35 dpi, small intestine and masseter muscles were collected from infected mice and blank control mice. Tissues were fixed in 4% paraformaldehyde for 24 h, embedded in paraffin wax, and cut into 3-µm-thick tissue cross-sections. Deparaffinized sections were stained using hematoxylin and eosin (HE) stain and periodic acid Schiff reagent (PAS; Baso, Zhuhai, China) [14,26]. Gut mucosal morphology was examined under light microscopy, the width of enteral villi and the number of goblet cells per high-power field (400×) were quantified. On muscle sections, the number of encapsulated larvae per low-power field (100×) and inflammatory cell infiltration (eosinophils, neutrophils, and lymphocytes) per high-power field (400×) were counted as previously described [38,39].

### Measurement of intestinal permeability

Intestinal TJs protein expression levels are directly correlated with intestinal permeability, which is typically determined in vivo using 4 kDa FITC-dextran (FD-4) [18,20]. To evaluate the intestinal permeability changes following subcutaneous immunization with rTsSPc, the mice were fasted overnight at 7 dpi. A 50 mg/ml FD-4 solution was prepared by dissolving 4 kDa FD-4 powders in sterile PBS, and 100 µl FD-4 was intragastrically administered to each mouse, then water access was restored. Four hours later, blood was collected from the orbital sinus, and plasma was separated by centrifugation at 4 °C and 1000 *g* for 10 min. The collected plasma was diluted with PBS (1:100) and added to a 96-well ELISA plate. Fluorescence intensity was measured using a microplate reader at excitation and emission wavelengths of 485 nm and 520 nm, respectively [17,40].

### Flow cytometry of murine peritoneal macrophages

Five mice of each group were euthanized at 2 weeks after final immunization and 1 week post infection, subsequently immersed in 75% alcohol for 2 min [41]. A total of 10 ml sterile PBS was injected intraperitoneally, followed by gentle pressure on peritoneal wall for 5 min before slowly aspirating the fluid into 15 ml centrifuge tubes [42,43]. Peritoneal cells were centrifuged at 500 *g* for 10 min at 4°C, and the supernatant was discarded. The cell pellet was resuspended in complete DMEM culture medium and seeded at a density of 5 × 10^6^ cells per well in 6-well plates. After incubation for 4 h, non-adherent cells were removed by medium replacement, and cells were washed three times with PBS. The resulting adherent cells were defined as peritoneal macrophages and continued to be cultured in complete DMEM. To analyze macrophage polarization states, cells were also treated with LPS (M1) or IL-4 (M2). Macrophage surface markers were analyzed via flow cytometry using FITC-F4/80, PerCP-Cyanine5.5-CD11b and PE-CD86, while intracellular expression of APC-CD206 was detected after permeabilization [44,45].

### qPCR and ELISA measurement of macrophage M1/M2 factors

Peritoneal macrophages were collected from three groups of mice at 2 weeks post immunization and 1 week after infection. Total RNA was extracted, and cDNA was synthesized via reverse transcription. qPCR was performed using the synthesized cDNA as a template to assess transcription levels of M1 (CD86, iNOS, TNF-α and IL-6) and M2-related factors (CD206, Arg1, TGF-β and IL-10). LPS (200ng/ml) served as the M1 control, while IL-4 (20ng/ml) served as the M2 control. DMEM served as the negative control. β-actin was used as the internal reference gene. Gene transcription levels were analyzed by comparing Ct values using the 2^−ΔΔCt^ method, and all assays were performed with triplicate [32], and qPCR primers used in this study are listed in Table 1. Additionally, macrophage culture supernatant was centrifuged at 1000 *g*, 4 °C for 10 min, and macrophage-secreted inflammatory cytokines (TNF-α, IL-6, TGF-β and IL-10) were assayed by a sandwich ELISA kit (Biolegend, USA [26,38]. Cytokine concentration was showed as pictograms per milliliter (pg/ml).

Western blot analysis was performed to measure the expression levels of M1 (iNOS) and M2-related factors (Arg1). Primary antibodies consisted of rabbit anti-iNOS (1:1000, Abcam) and mouse anti-Arg1 (1:5000, Proteintech), which were incubated at 4°C overnight [46]. Secondary antibodies included goat anti-rabbit IgG (1:5000, Upingbio) and goat anti-mouse IgG (1:5000, Upingbio), incubated at 37°C for 1 h. β-actin served as the internal reference, with three biological replicates per group [47]. Protein band intensities were analyzed using ImageJ software, and statistical analysis was conducted based on the ratio of target protein gray values to β-actin gray values.

### Antibody-dependent cell-mediated cytotoxicity (ADCC) assay

Specific antibody-mediated cytotoxicity killing *T. spiralis* NBL was assessed as previously described [45]. Briefly, female adult worms were collected at 6 dpi, cultivated in DMEM containing 10%FBS (Gibco) at 37 °C under 5% CO₂ for 24 h, and NBL were recovered. A total of 100 NBL were co-cultured with 3 × 10⁵ murine peritoneal exudate cells (PECs) in a 48-well plate. The cultures were then incubated with serial dilutions (1:50–1:800) of immune sera from rTsSPc, ISA 201 and PBS groups, *T. spiralis* infection sera were used as a positive control. After incubation at 37 °C for 72 h, NBL viability was determined based on larval morphology and motility [48]. Live larvae exhibited active movement, whereas dead larvae appeared rigid and immobile. Cytotoxicity was defined as the percentage of dead larvae relative to the total number of larvae observed in each well [49].

### Statistical analysis

Data were analyzed using GraphPad Prism 9.5.0 software. Results are expressed as the mean ± standard deviation (SD). The normality and homogeneity of the datum variance were evaluated by using the Shapiro-Wilk test and Levene’s test, respectively. Statistical analysis was performed using one-way analysis of variance (ANOVA) and t-tests. A *P* value less than 0.05 was considered statistically significant.

## Results

### Anti-rTsSPc antibodies in immunized mice

Serum anti-rTsSPc IgG titers were determined by ELISA at two weeks after the final vaccination. The results demonstrated that anti-rTsSPc IgG levels in vaccinated mice were significantly elevated compared to the pre-immunization levels (*F* = 17.22, *P* < 0.0001). The specific IgG titer reached 1:10^5^ at two weeks following final immunization (Fig 1B), indicating that rTsSPc had good immunogenicity. However, no anti-rTsSPc IgG responses were detected in mice immunized with ISA201 and PBS alone.

The kinetics of serum anti-rTsSPc antibodies were assayed by ELISA, the results showed that compared with the PBS group, the levels of anti-rTsSPc IgG in the rTsSPc-immunized group were significantly elevated at weeks 2, 4, 6, 7, 9 and 11 post immunization (*F*_2W_ = 47.13, *F*_4W_ = 4775, *F*_6W_ = 596.3, *F*_7W_ = 396.4, *F*_9W_ = 169, *F*_11W_ = 317; *P* < 0.0001). Furthermore, the IgG1 levels were significantly higher than IgG2a levels at various times after immunization (*t*_2W_ = 85.1, *t*_4W_ = 9.741, *t*_6W_ = 27.94; *t*_7W_ = 37.28, *t*_9W_ = 11.87, *t*_11W_ = 8.479; *P* < 0.0001), indica*t*ing that rTsSPc vaccination induced a mixed Th1/Th2 immune response with a Th2 dominance. Additionally, anti-rTsSPc IgA in the sera of immunized mice was also measured, and the results demonstrated that IgA levels were significantly increased at weeks 2, 4, 6, 7, 9 and 11 compared to the PBS control group (*F*_2W_ = 1768, *F*_4W_ = 287.4, *F*_6W_ = 136.7, *F*_7W_ = 256.8, *F*_9W_ = 97.95, *F*_11W_ = 61.58; *P* < 0.0001) (Fig 1C). After challenge infection, both ISA201 and PBS groups exhibited significantly elevated anti-rTsSPc IgG and IgA levels compared to pre-infection levels. These findings showed that specific IgG, IgG1/IgG2a and IgA levels in the rTsSPc group were gradually increased after immunization, and further elevated following challenge, indicated that rTsSPc vaccination elicited an evident antibody response.

Intestinal mucosal total and rTsSPc-specific sIgA responses were measured by sandwich and indirect ELISA. The results showed that there were no significant differences of total and specific sIgA levels among the rTsSPc, ISA201 and PBS groups before immunization (*P* > 0.05). But at two weeks after the final immunization, total sIgA levels in intestinal fluid of rTsSPc-immunized mice were significantly elevated compared to the PBS group (*F* = 60.58, *P* < 0.0001); specific sIgA levels in the rTsSPc group were also significantly higher than the PBS group (*F* = 48.04, *P* < 0.0001) (Fig 1D). No specific sIgA responses were detected in only ISA201 or PBS alone group. Moreover, at 7 and 11 weeks after immunization (e.g., 1 and 5 weeks after infection), both ISA201 and PBS groups exhibited significantly increased total and rTsSPc-specific sIgA levels compared to pre-infection levels (1 week after infection: total sIgA: *t*_ISA201_ = 6.207; *P* = 0.0003; *t*_PBS_ = 8.453; *P* < 0.0001; specific sIgA: *t*_ISA201_ = 3.180; *P* = 0.013; *t*_PBS_ = 4.858; *P* = 0.0013; 5 weeks af*t*er infection: total sIgA: *t*_ISA201_ = 5.746; *P* = 0.0004; *t*_PBS_ = 10.07; *P* < 0.0001; specific sIgA: *t*_ISA201_ = 13.96; *P* < 0.0001; *t*_PBS_ = 30.05; *P* < 0.0001). These results indicated tha*t* rTsSPc immunization induced a strong specific gut mucosal sIgA response.

### Analysis of cellular immune response type (Th1/Th2) of immunized mice

**Fig 2:**
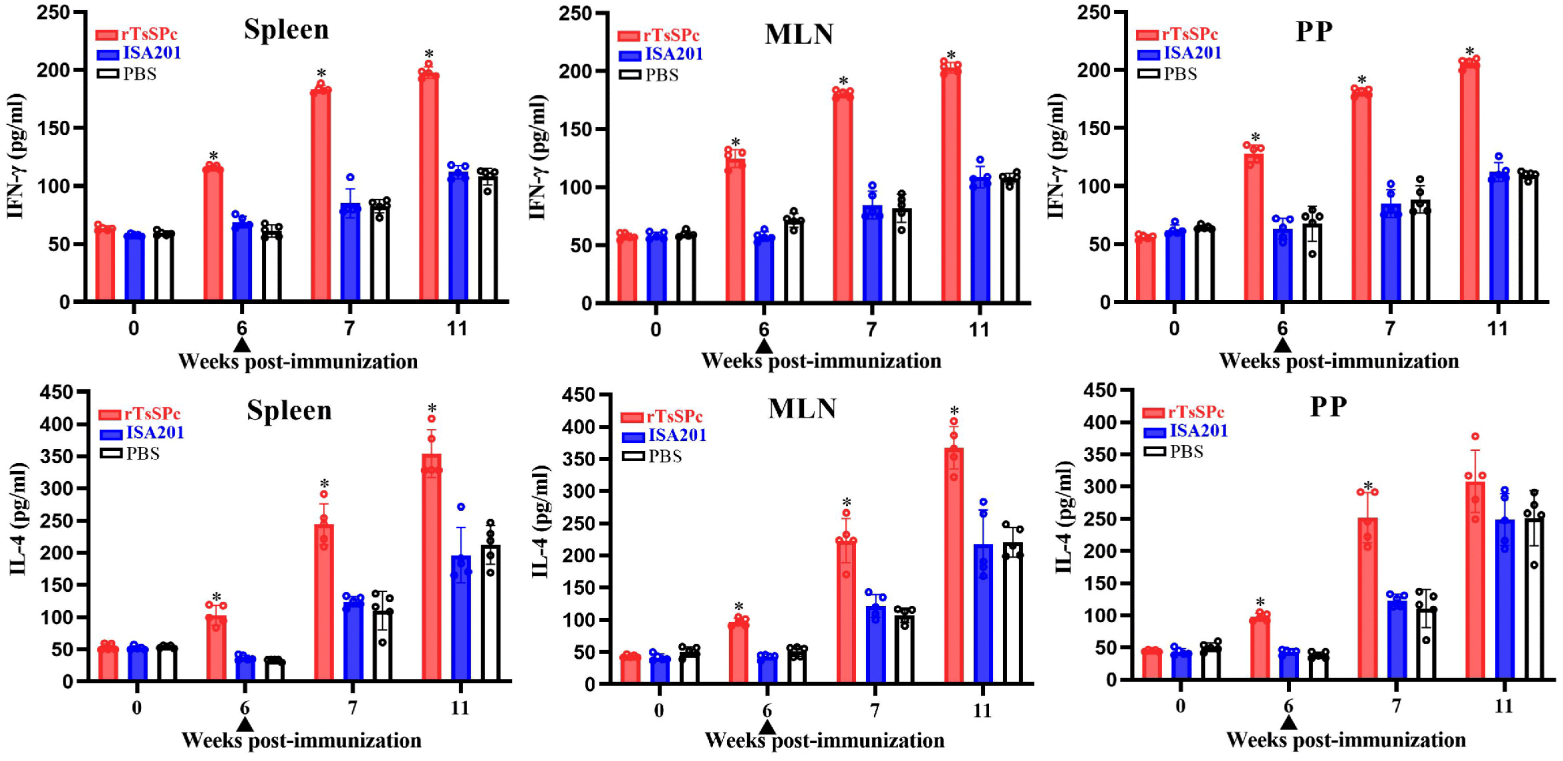
Cytokine secretion profiles from spleen, mesenteric lymph nodes (MLN) and Peyer’s patches (PP) of rTsSPc-immunized mice at different times after vaccination. Concentrations of IFN-γ and IL-4 were quantified in culture supernatants following stimulation with 10 µg/ml rTsSPc at 37 °C and 5% CO_2_ for 72 h. Data represent the mean picograms per milliliter (pg/ml) ± SD (n = 5). All samples were assayed in duplicate. *P < 0.05 compared to the PBS control group.

To investigate the cellular immune response type (Th1/Th2) induced by rTsSPc vaccination, spleen, MLN and Peyer’s patch were isolated from immunized BALB/c mice. Their cells were stimulated with rTsSPc, and the cytokine secretion levels in culture supernatants were detected by ELISA. The results showed that the levels of Th1 (IFN-γ) and Th2 cytokine (IL-4) of three groups had no significant differences prior to immunization (*P* > 0.05). At 6 weeks after immunization, the levels of IFN-γ and IL-4 secreted by spleen cells in the rTsSPc group were significantly elevated compared to the PBS group (*F*_IFN-γ_ = 233.5, *F*_IL-4_ = 89.09, *P* < 0.0001). Furthermore, the expression levels of IFN-γ and IL-4 were further increased at 7 weeks after immunization (1 week after challenge; *F*_IFN-γ_ = 247.5, *F*_IL-4_ = 41.85, *P* < 0.0001). And the levels of IFN-γ and IL-4 of spleen cells were further elevated at 11 weeks after immunization (5 weeks after challenge; *F*_IFN-γ_ = 351.5, *F*_IL-4_ = 27.17, *P* < 0.0001). The levels of IFN-γ and IL-4 secreted by MLN cells in the rTsSPc group were also significantly higher than the PBS group (6 weeks: *F*_IFN-γ_ = 167.7, *F*_IL-4_ = 252.8, *P* < 0.0001; 11 weeks: *F*_IFN-γ_ = 360.8, *F*_IL-4_ = 25.05, *P* < 0.0001). Similarly, the levels of IFN-γ and IL-4 secreted by PP cells in the rTsSPc group were significantly higher than the PBS group at 6 weeks post immunization (*F*_IFN-γ_ = 54.52, *F*_IL-4_ = 220.9, *P* < 0.0001); but at 11 weeks post immunization, only IFN-γ levels secreted by PP cells in the rTsSPc group was higher the PBS group(*F*_IFN-γ_ = 479.8, *P* < 0.0001; *F*_IL-4_ = 2.926, *P* > 0.05) (Fig 2). These findings indicate that subcutaneous immunization with rTsSPc not only induced systemic (spleen) cellular immunity, but also elicited gut local (MLN and PP) mucosal cellular immunity, resulting in a mixed Th1/Th2 immune response.

### rTsSPc immunization increased TJs expression in infected mice

**Fig 3:**
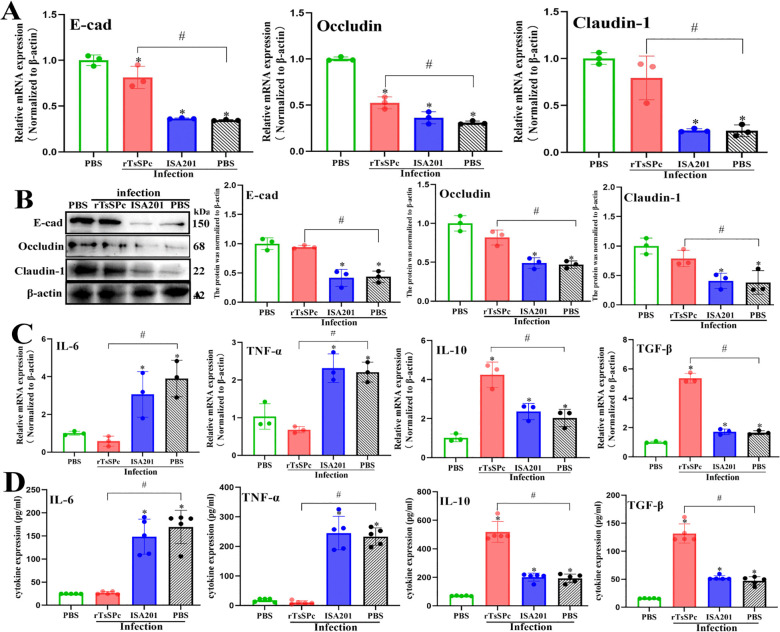
qPCR, Western blot and ELISA determination of mRNA and protein expression of gut TJs and cytokines in immunized mice at 7 days post infection. **A**: qPCR analysis of mRNA expression levels of TJs (E-cad, Occludin and Claudin-1) in intestines of immunized mice (n = 3). **B:** Western blot analysis of TJs protein expression levels in intestines of immunized mice (n = 3). **C:** qPCR analysis of mRNA levels of TNF-α, IL-6, TGF-β and IL-10 in intestinal tissues (n = 3). β-actin was used as an internal control. Each experiment was repeated three times. **D:** ELISA analysis of protein expression levels of TNF-α, IL-6, TGF-β and IL-10 in intestinal lavage fluids (n = 5). *P < 0.05 compared with the uninfected PBS group; ^#^ *P* < 0.05 compared with the infected PBS group.

qPCR results showed that at 7 dpi, the transcript levels of E-cad, Occludin and Claudin-1 in three group of rTsSPc, ISA 201 and PBS groups after infection were obviously declined compared to the uninfected PBS group (*F*_E-cad_ = 71.39, *F*_Occludin_ = 128.7, *F*_Claudin-1_ = 29.74, *P* < 0.001), but their transcript levels in the rTsSPc group were significantly higher than the infected PBS group (*t*_E-cad_ = 6.410, *P* = 0.003; *t*_Occludin_ = 5.562, *P* = 0.0051; *t*_Claudin-1_ = 4.041, *P* = 0.0156) (Fig 3A). Western blot results revealed that at 7 dpi, the protein expression levels of E-cad, Occludin and Claudin-1 in the three group were evidently lower than the uninfected PBS group (*F*_E-cad_ = 29.06, *F*_Occludin_ = 31.13, *F*_Claudin-1_ = 11.52, *P* < 0.01), but their protein levels in the rTsSPc group were significantly higher than the infected PBS group (*t*_E-cad_ = 8.741, *P* = 0.0009; *t*_Occludin_ = 5.660, *P* = 0.0048; *t*_Claudin-1_ = 2.882, *P* = 0.0449) (Fig 3B). These findings indica*t*ed that rTsSPc immunization counteracted and restored the TJs protein downregulation caused by *T. spiralis* infection, suggesting that rTsSPc vaccination enhanced murine intestinal epithelial integrity and barrier function after infection.

### Cytokine transcription and expression levels in intestinal mucosa of infected mice

qPCR results showed that compared with the infected PBS group, the transcription levels of pro-inflammatory cytokines (TNF-α and IL-6) in murine intestinal mucosa of the rTsSPc group were significantly decreased (*t*_TNF-α_ = 9.553, *P* = 0.0007; *t*_IL-6_ = 5.755, *P* = 0.0045), while the transcription levels of anti-inflammatory cytokines (TGF-β and IL-10) were significantly increased (*t*_TGF-β_ = 17.87, *P* < 0.0001; *t*_IL-10_ = 4.882, *P* = 0.0081) (Fig 3C). ELISA results revealed that the expression levels of pro-inflammatory cytokines (TNF-α and IL-6) in intestinal lavage fluids of the rTsSPc-immunized mice were significantly lower than the infected PBS group (*t*_TNF-α_ = 16.70, *t*_IL-6_ = 8.820, *P* < 0.0001), whereas the expression levels of anti-inflammatory cytokines (TGF-β and IL-10) were significantly higher than the infected PBS group (*t*_TGF-β_ = 10.19, *t*_IL-10_ = 9.253, *P* < 0.0001) (Fig 3D). These results indicated that rTsSPc immunization significantly reduced the transcription and secretion levels of pro-inflammatory cytokines (TNF-α and IL-6), but increased expression of anti-inflammatory cytokines (TGF-β and IL-10), suggesting that rTsSPc immunization alleviated intestinal inflammation.

### rTsSPc immunizations blocked the binding between parasite-derived TsSPc and gut epithelial RACK1 receptor

**Fig 4:**
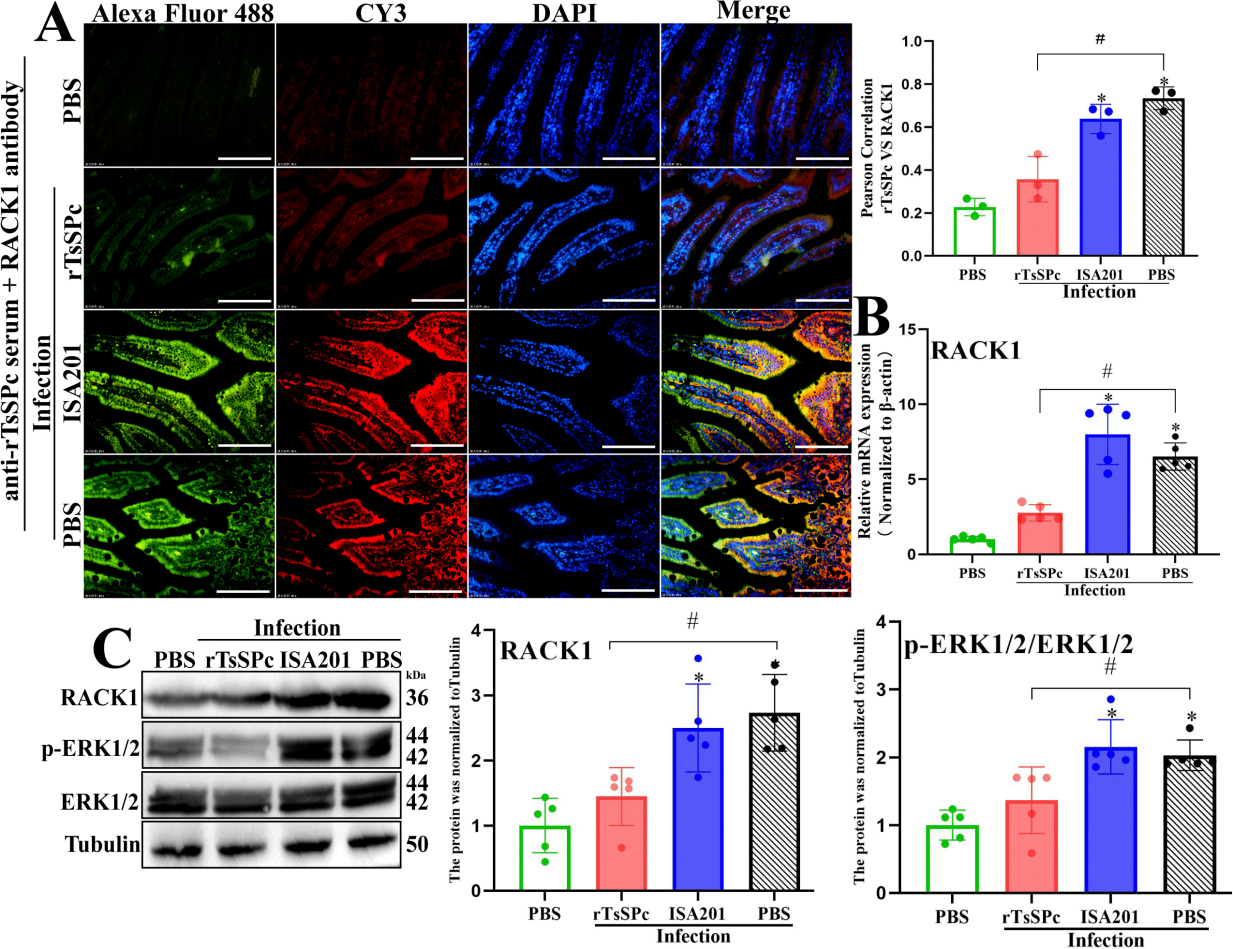
rTsSPc immunizations blocked the binding of parasite-derived TsSPc and gut RACK1 receptor after infection. **A:** IFA co-localization of TsSPc and RACK1 on gut epithelium. Small intestinal sections were incubated with anti-rTsSPc serum, followed by Alexa Fluor 488-conjugated goat anti-mouse IgG. Intestinal sections were incubated with anti-RACK1 antibody and stained with Cy3-conjugated goat anti-rabbit IgG for detecting gut epithelial RACK1 receptors. Cellular nuclei were counterstained blue with DAPI. Scale bar: 100 μm. **B:** qPCR analysis of mRNA expression levels of RACK1. **C:** Western blot analysis of protein expression levels of RACK1 and p-ERK1/2/ERK1/2. *P < 0.05 compared to the uninfected PBS group; ^#^*P* < 0.05 compared with the infected PBS group.

IFA results showed that at 7 dpi, TsSPc-specific green fluorescence was not detected in intestinal epithelium of the rTsSPc group. But green fluorescence was observed in both the ISA201 and PBS groups. Anti-RACK1 antibody identified the natural RACK1 receptor in intestinal epithelium as red fluorescence. After being merged, compared with the infected PBS group, the intensity of yellow fluorescence resulting from co-localization between rTsSPc and RACK1 in intestinal epithelium was significantly reduced in the rTsSPc group. Pearson correlation coefficients for the rTsSPc, ISA201, infected PBS and uninfected PBS groups were 0.357, 0.638, 0.734, and 0.227, respectively. Pearson correlation coefficient of the rTsSPc group was obviously lower than the infected PBS group (*F =* 33.17*, P <* 0.0001) (Fig 4A).

qPCR results showed that at 7 dpi, RACK1 transcription level in the rTsSPc group was obviously lower than the infected PBS group (*t* = 7.925, *P* < 0.0001), it was not statistically different from the uninfected PBS group (*P* > 0.05) (Fig 4B). Western blot analysis revealed that at 7 dpi, protein expression levels of RACK1 and phosphorylated ERK1/2 (p-ERK1/2) in the rTsSPc group were significantly lower than the infected PBS group (*t*_RACK1_ = 3.897, *P* = 0.0046; *t*_p-ERK1/2/ERK1/2_ = 2.737, *P* = 0.0256), but they had no statistical difference from the uninfected PBS group (*P* > 0.05). Moreover, expression levels of RACK1 and p-ERK1/2 in ISA201 and infected PBS group were markedly elevated compared with the uninfected PBS group (*F*_RACK1_ = 11.74, *P* = 0.0003; *F*_p-ERK1/2/ERK1/2_ = 12.01, *P* = 0.0002) (Fig 4C).

These findings suggested that anti-rTsSPc antibodies produced by rTsSPc immunization might neutralize the parasite-derived TsSPc and block its binding to gut epithelial RACK1 receptor, subsequently prevent the activation of MAPK/ERK1/2 pathway and destruction of gut epithelial integrity, and ultimately impede the parasite invasion.

### Intestinal and muscle histopathological change in infected mice

**Fig 5:**
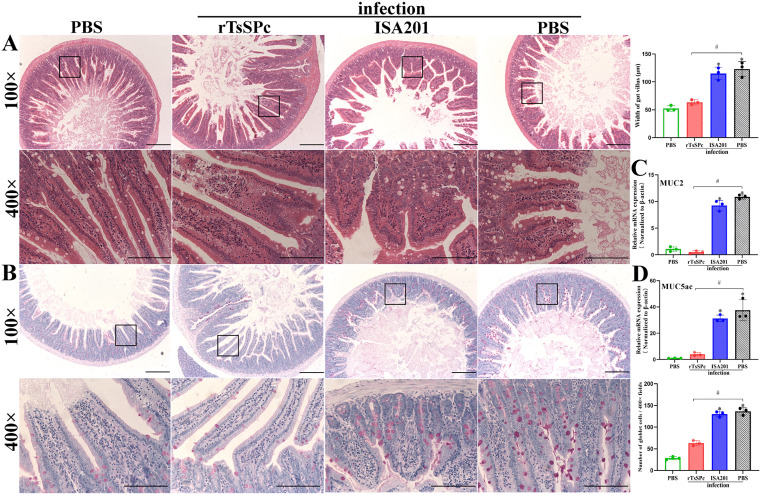
Histopathological changes in murine intestinal tissues at 7 days post infection. **A:** HE staining of duodenum section under a microscope. The pathological alterations were significantly alleviated in the rTsSPc group compared to ISA201 and PBS control groups. In contrast, severe inflammation was observed in the intestinal mucosa of the ISA201 and PBS groups, as characterized by obvious villus shortening and edema. Intestinal villus width of each group was measured (n = 3). **B:** PAS staining of duodenum tissue sections under a microscope. Compared to the ISA201 and PBS control groups, the rTsSPc-immunized group exhibited a significant reduction of goblet cell number. **C:** Relative expression levels of Muc2 mRNA. **D:** Relative expression levels of Muc5ac mRNA. Scale bar = 200 μm. *P < 0.05 compared to the uninfected PBS group; ^#^*P* < 0.05 compared with the infected PBS group.

Intestinal sections HE staining revealed that both ISA201 and infected PBS groups exhibited severe damage to intestinal villi with significant edema at 7 dpi. In contrast, the rTsSPc-immunized mice only showed mild inflammatory responses, with slight villous edema and relatively intact villous architecture. Compared to the infected PBS group, the villus width of the rTsSPc group was significantly reduced (*F* = 45.16, *P* < 0.0001) (Fig 5A). PAS staining showed that the number of goblet cells was distinctly increased and their volume became larger in the ISA201 and PBS groups, whereas the rTsSPc group exhibited a clear decrease of goblet cell numbers and smaller cell size. Compared to the infected PBS group, the number of goblet cells in the rTsSPc group was significantly reduced (*F* = 211.3, *P* < 0.0001) (Fig 5B). Furthermore, qPCR results showed that transcription levels of Muc2 and Muc5ac of the rTsSPc group were significantly lower than the infected PBS group (*F*_Muc2_ = 259.2, *F*_Muc5ac_ = 58.07, *P* < 0.0001) (Fig 5C, 5D). These findings suggested that rTsSPc immunization effectively inhibited the *T. spiralis* larva invasion of intestinal mucosa, alleviated intestinal inflammation, and decreased mucin expression in intestinal mucosa.

**Fig 6:**
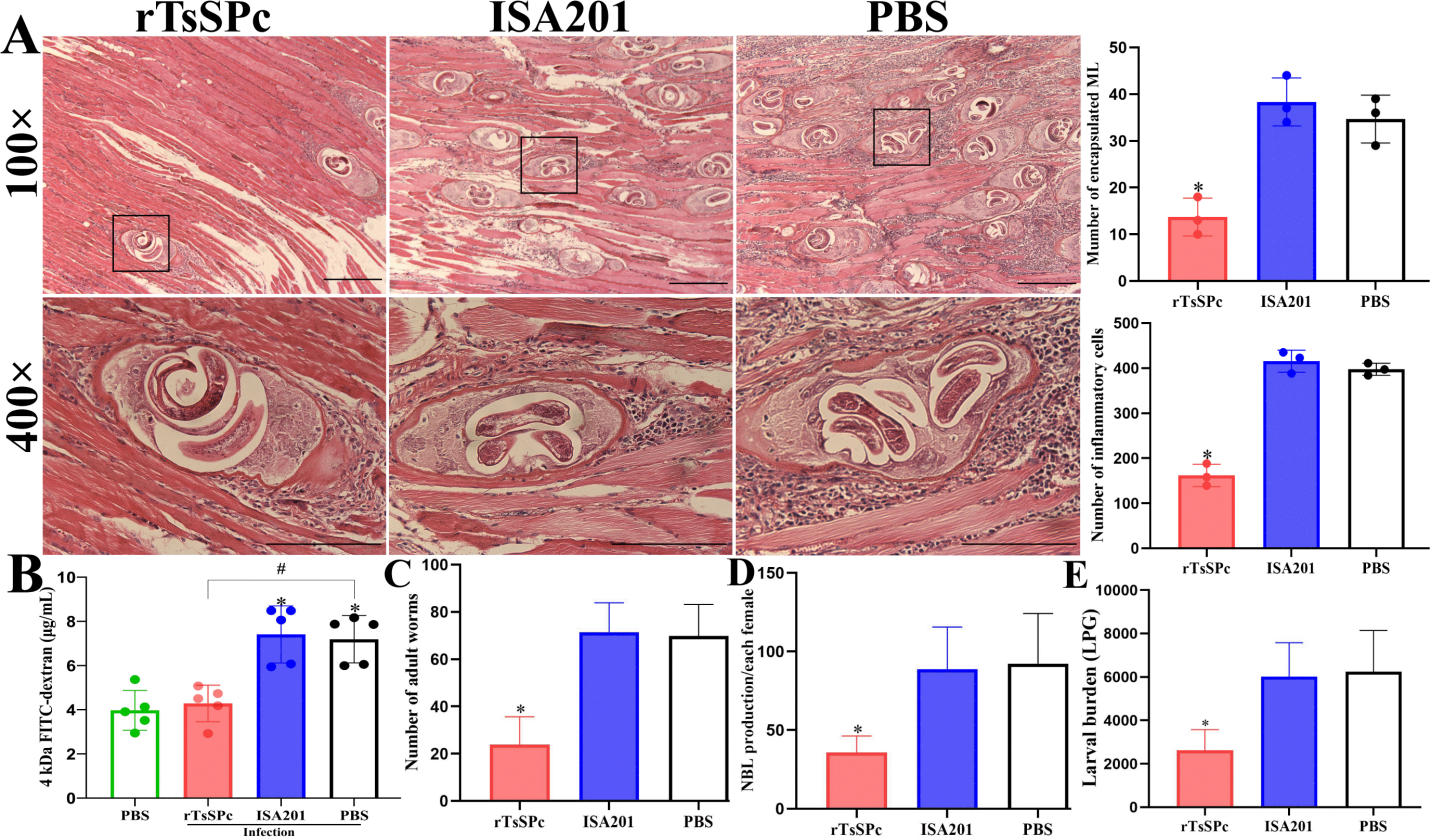
Histopathology of murine diaphragm, gut permeability and worm burdens in infected mice. **A:** HE staining of diaphragm muscle sections under a microscope. The rTsSPc-immunized group exhibited mild inflammatory reactions and fewer larvae compared to the ISA201 and PBS control groups. Scale bar = 200 μm. Quantification of encapsulated muscle larvae (ML) and inflammatory infiltration around larval capsules was performed in various groups. **B:** gut permeability of various groups of mice after infection. The passage of FD-4 through the gut was significantly reduced in rTsSPc group compared to the infected PBS group following *T. spiralis* infection (n = 5). **C:** Intestinal adult worm burden (n = 10). **D:** Newborn larvae (NBL) production per female worm (n = 30). **E:** Muscle larval burden (LPG, n = 10). Data are expressed as mean ± SD. *P < 0.05 compared to the PBS group. ^#^ *P* < 0.05 compared to infected PBS group.

At 35 dpi, muscle sectional HE staining revealed obvious inflammation around the encapsulated larvae in the diaphragm of the PBS and ISA201 groups, characterized by massive infiltration of inflammatory cells. However, inflammatory infiltration around the encapsulated larvae in the rTsSPc group was relieved (Fig 6A). Compared to the PBS group, the number of the larvae within collagen capsules of the rTsSPc group was significantly reduced (*F* = 23.11, *P* = 0.0015). The inflammatory infiltration surrounding collagen capsules in the rTsSPc group was also significantly lower than the PBS groups (*F* = 129.8, *P* < 0.0001).

### rTsSPc immunization decreased gut permeability and reduced worm burdens

To evaluate intestinal permeability following rTsSPc immunization, mice were challenged with 300 *T. spiralis* ML two weeks after the final immunization. At 7 dpi, mice were orally administered with FD-4, and plasma was collected to be ascertained 4 hours later. The results showed that compared to the infected PBS group, the FD-4 content in the rTsSPc group was significantly reduced by 40.43% (*F* = 15.66, *P* < 0.0001) (Fig 6B). These findings indicated that rTsSPc immunization significantly declined the gut permeability increased from *T. spiralis* infection, thereby enhancing intestinal mucosal epithelial integrity. This protective effect may be associated with the rTsSPc immunization to prevent larval invasion and reduce intestinal inflammation.

At 7 dpi, AWs were collected from the intestines of ten mice per group and enumerated. The results showed that compared to the PBS control group, intestinal adult burden in the rTsSPc group was significantly reduced, with a 65.7% reduction of adult burden (*F* = 46.24, *P* < 0.0001) (Fig 6C). To assess female fecundity, three female worms from each group were randomly selected and cultured at 37 °C and 5% CO_2_ for 72 h, and the NBL were collected. The results revealed that the adult female reproductive capacity of the rTsSPc group was significantly lower than the PBS group, with a 61.13% reduction of female fecundity (*F* = 16.32, *P* < 0.0001) (Fig 6D). Furthermore, at 35 dpi, the muscle larval burden in the rTsSPc group was decreased with a reduction of 58.10% (*F* = 17.80, *P* < 0.0001) (Fig 6E). These findings demonstrated that rTsSPc immunization significantly reduced adult burden, hindered enteral worm growth and decreased female fecundity, thereby reduced muscle larval burden, indicating that rTsSPc vaccination elicited an obvious immune protection against *T. spiralis* infection.

### Polarization of peritoneal macrophages in rTsSPc-immunized mice

**Fig 7:**
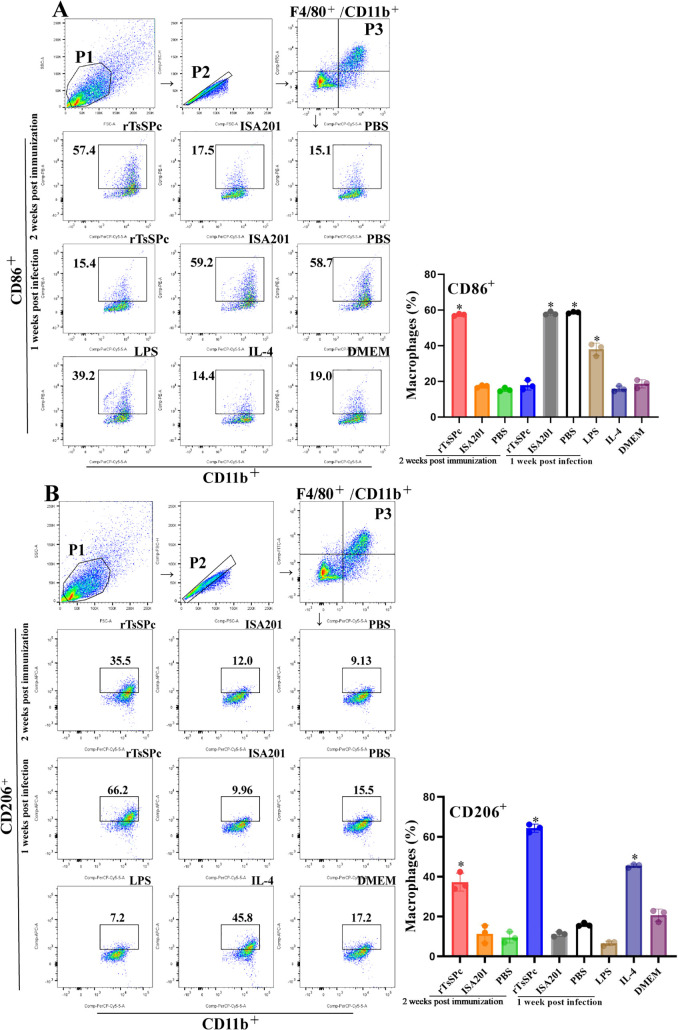
Flow cytometry of murine peritoneal macrophages after rTsSPc immunization and larval infection. **A:** Assessment of CD86 (M1) expression levels (n = 3). LPS (200 ng/ml) served as the positive control for M1 polarization. Macrophages were identified using F4/80^+^/CD11b^+^ markers. **B**: Assessment of CD206 (M2) expression levels. IL-4 (20 ng/ml) served as the positive control for M2 polarization. *P < 0.05 compared to the PBS group.

Flow cytometry was performed to evaluate macrophage polarization of immunized murine. Peritoneal macrophages were identified based on F4/80^+^/CD11b^+^ expression, with CD86^+^ cells defined as the M1 phenotype and CD206^+^ cells defined as the M2 phenotype. The results showed that compared to the PBS group, the expression levels of both CD86^+^ and CD206^+^ in peritoneal macrophages from the rTsSPc group were significantly increased at 2 weeks post-immunization (*F*_CD86_+ = 2581, *F*_CD206_+ = 45.65, *P* < 0.001) (Fig 7A). However, compared to the PBS group, CD86^+^ expression of the rTsSPc group at 1 week post-infection was significantly decreased(*F* = 566.5; *P* < 0.0001), but CD206^+^ levels were obviously increased (*F* = 1260, *P* < 0.0001) (Fig 7B). The data indicated that peritoneal macrophages of the rTsSPc group exhibited a mixed M1/M2 polarization pattern at 2 weeks after final immunization, whereas the macrophages displayed a M2 polarization at 1 week after infection.

### qPCR, ELISA and Western blotting of mRNA and protein expression level of M1/M2 factors in peritoneal macrophages

**Fig 8:**
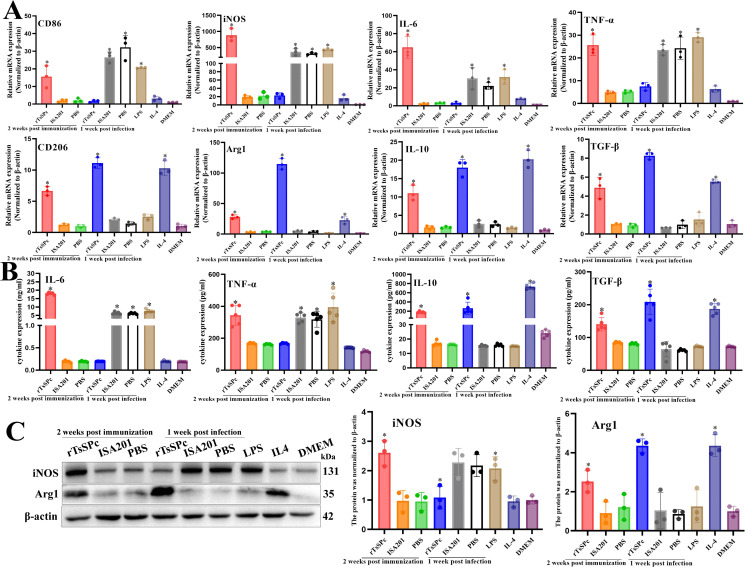
qPCR, ELISA and Western blotting of mRNA and protein expression levels of M1/M2-related factors in murine peritoneal macrophages. **A:** qPCR analysis of mRNA levels of M1 (CD86, iNOS, TNF-α and IL-6) and M2-related factors (CD206, Arg1, TGF-β and IL-10) in murine peritoneal macrophages (n = 3). **B:** ELISA measurement of macrophages-secreted cytokines (TNF-α, IL-6, TGF-β and IL-10) (n = 3). **C:** Western blotting of iNOS (M1) and Arg1 (M2) expression in murine peritoneal macrophages. iNOS and Arg1 protein expression levels of murine peritoneal macrophages were assayed at 2 weeks post immunization and 1 week after infection (n = 3). LPS (200 ng/ml) served as the M1 positive control, while IL-4 (20 ng/ml) served as the M2 positive control. *P < 0.05 compared to the PBS group or DMEM group.

qPCR results revealed that compared to the PBS group, peritoneal macrophages of the rTsSPc group exhibited significantly increased mRNA expression levels of both M1- and M2-associated genes at two weeks post last immunization (M1: *F*_CD86_ = 13.45, *F*_iNOS_ = 57.75, *F*_IL-6_ = 75.18, *F*_TNF-α_ = 60.88, *P* < 0.01; M2: *F*_CD206_ = 150.0, *F*_Arg1_ = 152.2, *F*_IL-10_ = 54.87, *F*_TGF-β_ = 36.66, *P* < 0.001). However, at one week post infection, the mRNA levels of M1-associated factors have a significant decrease compared to the PBS group (*F*_CD86_ = 43.77, *P* = 0.0003; *F*_iNOS_ = 25.44, *P* = 0.0012; *F*_IL-6_ = 11.17, *P* = 0.0095; *F*_TNF-α_ = 24.75, *P* = 0.0013), while the mRNA levels of M2-associated factors showed an obvious elevation (*F*_CD206_ = 311.6, *F*_Arg1_ = 420.7, *F*_IL-10_ = 113.3, *F*_TGF-β_ = 549.2, *P* < 0.0001) (Fig 8A).

ELISA results showed that compared with the PBS group, rTsSPc group exhibited significantly increased expression level of macrophage pro-inflammatory cytokines at 2 weeks after final immunization (*F*_IL-6_ = 3565, *F*_TNF-α_ = 40.88, *P* < 0.0001); simultaneously anti-inflammatory cytokine expression level was also significantly increased (*F*_IL-10_ = 409.1, *F*_TGF-β_ = 39.11, *P* < 0.0001). However, at 1 week post-infection, pro-inflammatory cytokine level of rTsSPc group was obviously declined compared to the PBS group (*F*_IL-6_ = 255.6, *F*_TNF-α_ = 37.86, *P* < 0.0001), but anti-inflammatory cytokine levels were evidently elevated (*F*_IL-10_ = 17.99, *F*_TGF-β_ = 51.86, *P* < 0.001) (Fig 8B).

Western blot results showed that compared to the PBS group, the expression levels of both iNOS and Arg1 in peritoneal macrophages from the rTsSPc group were significantly upregulated at 2 weeks post-immunization (*F*_iNOS_ = 19.97, *P* < 0.01; *F*_Arg1_ = 6.094, *P* = 0.0359). However, at 1 week post-infection, iNOS expression was obviously decreased (*F*_iNOS_ = 7.661, *P* = 0.0223), Arg1 expression was significantly increased (*F*_Arg1_ = 34.05, *P* < 0.001) (Fig 8C). No significant differences of iNOS and Arg1 expression between the PBS and ISA201 groups were observed at 2 weeks post-immunization and 1 week post-infection (*P* > 0.05).

These findings further confirmed that peritoneal macrophages from rTsSPc-immunized mice exhibited a mixed M1/M2 polarization phenotype at 2 weeks after immunization, but the macrophages displayed a M2 polarization at 1 week after infection.

### ADCC killing NBL

**Fig 9:**
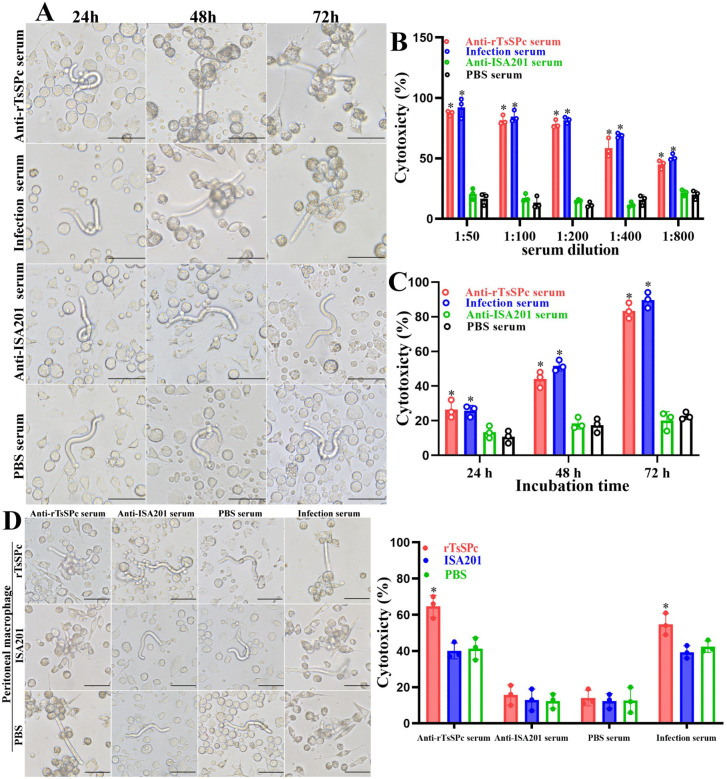
Anti-rTsSPc antibodies mediated ADCC of various macrophages killing NBL. **A:** Anti-rTsSPc serum mediated PECs adhesion to NBL and subsequent destruction of NBL. One hundred NBL were co-cultured for 72 h with normal murine PECs (3 × 10^5^) and different dilutions of anti-rTsSPc serum. Infection serum served as the positive control, while sera from ISA201 and PBS groups served as negative controls. **B:** Cytotoxicity was dependent on dose of anti-rTsSPc antibodies. **C: C**ytotoxicity was dependent on the culture time. **D:** Cytotoxicity of various groups of murine macrophage killing NBL via ADCC. One hundred newborn larvae were co-cultured for 48 h with peritoneal macrophages and immune sera collected from different groups of mice at 2 weeks post vaccination. When anti-rTsSPc serum or infection serum was used, macrophage cytotoxicity of the rTsSPc group was also significant higher than the PBS and ISA 201 groups. Scale bar = 50 μm. *P < 0.05 compared to the PBS group.

Anti-rTsSPc antibodies mediated the adhesion of PECs to the NBL and induced larval damage and death (Fig 9A). When 1:100 dilutions of anti-rTsSPc sera were co-cultured with normal murine PECs and NBL for 72 h, ADCC efficiently killed the NBL with a 81.68% cytotoxicity, which was significantly higher than the sera from the PBS group (*F* = 258.7, *P* < 0.0001) (Fig 9B). This cytotoxic effect exhibited a dose-dependent on immune serum dilution (*r* = 0.964, *P* < 0.0001). Furthermore, the cytotoxicity was increased gradually with prolonged culture time (*F*_24h_ = 13.03, *P* = 0.0019; *F*_48h_ = 65.26, *F*_72h_ = 302.8, *P* < 0.0001) (Fig 9C).

When various macrophages from three groups of mice at 2 weeks post immunization were co-cultured with the NBL and 1:100 dilutions of anti-rTsSPc sera for 48 h, the macrophages’ cytotoxicity of the rTsSPc, ISA201 and PBS was 64.67, 40.00 and 41.33%, respectively (*F* = 18.69, *P* = 0.0026); the cytotoxicity of macrophages from the rTsSPc group was obviously higher than the PBS group (*t*= 4.709, *P* = 0.0092) (Fig 9D). When the macrophages from three groups of mice were co-cultured with NBL and 1:100 dilutions of infection serum, the cytotoxicity were 54.67, 39.33 and 43.0%, respectively (*F* = 10.07, *P* = 0.0121). The infection serum-mediated cytotoxicity of macrophages from the rTsSPc group was also significant higher than the PBS group (*t* = 3.127, *P* = 0.0353). The results indicated that rTsSPc immunization obviously enhanced murine macrophages’ ADCC killing newborn larvae.

Additionally, the raw data of Figs 1-9 in this study were shown in S1 Table.

## Discussion

The TsSPc exists in the *T. spiralis* IIL surface and ES proteins; it is a secretory serine protease. TsSPc is expressed in all developmental stages and primarily localized in the cuticle and stichocytes of this nematode [13]. rTsSPc facilitated larval invasion of IECs; whereas anti-TsSPc immune sera and rTsSPc-specific dsRNA distinctly impeded larval invasion [16]. Previous studies showed that although rTsSPc was not capable of directly hydrolyzing gut epithelial TJs proteins, it could bind to gut epithelial receptor RACK1, activated MAPK/ERK1/2 signal pathway, declined the expression levels of TJs proteins and disrupted gut epithelial integrity, consequently promoted *T. spiralis* larval invasion of gut mucosa [17]. Moreover, rTsSPc specifically bound with gut epithelial cytokeratin 8 (CK8) receptors and activated the RhoA/ROCK1 pathway, also bound and interacted with enteral receptor phosphoglycerate mutase family member 5 (PGAM5) to activate apoptotic pathway, down-regulated the TJs expression, enlarged intestinal permeability and disrupted gut epithelial integrity, and accelerated larval invasion [18–20]. Collectively, these findings further suggested that TsSPc disrupted gut epithelial integrity through a synergistic mechanism of activating various receptors and signal pathways, and it could be considered as a candidate vaccine target for preventing *T. spiralis* infection.

To further evaluate the immune protective efficacy of rTsSPc, BALB/c mice were subcutaneously immunized with rTsSPc emulsified in ISA201 adjuvant. The results revealed that rTsSPc immunization elicited strong systemic and gut local mucosal humoral immune responses. At two weeks after the final immunization, serum anti-rTsSPc IgG titer reached 1:10⁵, and IgG1/IgG2a levels were significantly elevated, indicating that rTsSPc elicited a mixed Th1/Th2 immune response with Th2 predominance. The result was consistent with the immunological characteristics of the helminth-derived vaccine candidates [6,14,21,23–27]. The mixed Th1/Th2 immunity played a key role against *T. spiralis* infection. Concomitantly, the significant upregulation of intestinal sIgA in rTsSPc-immunized mice underscored the induction of local mucosal immunity, which is critical for blocking the *T. spiralis* IIL worm adhesion and invasion of intestinal epithelium. In addition, the secretion levels of Th1 cytokine IFN-γ and Th2 cytokine IL-4 in splenocytes, MLNs and PP cells were significantly increased at 6 weeks post-immunization and further elevated following challenge infection, suggesting that rTsSPc immunization also triggered an evident cellular immunity. As the core organs of intestinal mucosal immunity, the high levels of IL-4 secretion in MLNs and PPs indicated that rTsSPc not only induced systemic Th2 responses but also specifically elicited local intestinal mucosal Th2 immunity [23].

Most infective pathogens invade the host through mucosal surfaces, and sIgA is the first line protection barrier at these entrances [6]. Intestinal mucosal sIgA is a central effector molecule of mucosal immune system, exerts the key protective effects against intestinal infection via multiple mechanisms: directly targeting the pathogens, enhancing intestinal mucosal barrier function, and synergistically regulating the local immune microenvironment [50]. The sIgA plays an important role in mucosal defense and could block the parasite invasion of enteral epithelia [21,38]. The sIgA against *T. spiralis* IIL and adult epi-cuticle surface antigens promoted the worm expulsion from the gut, passive transfer of native mice with anti-*Trichinella* antibody IgA produced a 95% protection against challenge [51]. The sIgA is dependent of Th2 immunity; especially IL-4 is the primary cytokine enhancing IgA response. Our results also suggested that high levels of IL-4 strengthened enteral sIgA response [25]. Furthermore, enteral sIgA also decreased the reproductive capacity of intestinal female *T. spiralis* adults. Our results showed that female fecundity of rTsSPc- vaccinated mice was also significantly lower than the ISA 201 and PBS groups. The levels of total sIgA and rTsSPc-specific sIgA in enteral fluid of rTsSPc-immunized mice were significantly higher than those in the PBS group and further increased at post-infection, indicating that the Th2 response activated intestinal mucosal immune system to induce a more sIgA secretion; sIgA bound to intestinal *T. spiralis* larvae and intercepted worm adhesion and invasion of intestinal mucosa, and directly resulting in a 65.7% reduction of intestinal adult burden in the rTsSPc immunization group [26]. The mixed Th1/Th2 response integrated the pro-inflammatory and cytotoxic effects of Th1 immunity with the mucosal barrier enhancement and helminth expulsion effects of Th2 immunity.

A core objective of parasite vaccine development is to reduce parasite burden and mitigate pathological damage caused by parasite infection [52]. rTsSPc immunization exhibited significant immune protective efficacy against *T. spiralis* infection: the rTsSPc immunization group showed a 65.7% reduction of enteral adult burden and a 58.10% reduction of muscle larval burden, it is obviously superior to previous single vaccine molecule [14,23,27]. The marked decrease of female fecundity in rTsSPc immune group was also particularly notable, as it directly abrogated the NBL production and dissemination, thereby interrupting the parasite’s life cycle at the critical early stage of infection. Additionally, secretion levels of pro-inflammatory cytokines (TNF-α and IL-6) in intestinal fluids of the rTsSPc-immunized mice were significantly reduced, while anti-inflammatory cytokines (IL-10 and TGF-β) were significantly increased. IL-10 inhibits macrophage M1 polarization and reduces pro-inflammatory cytokine release, whereas TGF-β promotes intestinal mucosal repair and immune tolerance, preventing excessive inflammation-induced tissue damage [53]. Histopathological analysis showed that rTsSPc-immunized mice exhibited obviously attenuated intestinal villus edema and preserved villus structure. At 35 dpi, diaphragm sections from the PBS and ISA201 groups showed extensive inflammatory infiltration around larval capsules, while the rTsSPc-immunized group displayed not only a significantly reduced number of encapsulated larvae but also a markedly diminished inflammatory infiltration around larvae. These findings indicated that rTsSPc immunization not only reduced intestinal adult worm burden and female fecundity, but also regulated the systemic inflammatory microenvironment to suppress pathological inflammatory responses of muscle tissue, alleviating the infection-induced tissue damage, highlighting the role of Th2-mediated anti-inflammatory regulation in reducing both parasite burden and infection-associated tissue injury [54].

Mucus (predominantly Muc2 and Muc5ac) are the key functional proteins secreted by intestinal goblet cells, and they form a mucus layer that constitutes the first physical barrier of intestinal mucosa and participate in regulating intestinal inflammation and pathogen clearance [40]. In infected PBS and ISA201 groups, goblet cell number was significantly increased with enlarged volume, and Muc2/Muc5ac transcription levels were significantly higher than the rTsSPc-immunized group at one week post infection. This phenomenon may represent a host stress-induced defensive response to parasitic invasion: excessive mucus secretion might provide a cryptic niche for larvae to evade host immune attack [55]. The mucus main component is the mucin, a glycoprotein secreted by goblet cells which assembles becoming a sticky and elastic gelatinous monolayer [56]. Intestinal helminth infection usually leads to mucus layer thickening. The increased gut mucus wraps around intestinal *T. spiralis* worms and limits their activity, and impedes *T. spiralis* growth and survival in the gut [57]. Moreover, rTsSPc-induced intestinal mucosal sIgA and serum-specific antibodies directly targeted and neutralized the larval-secreted TsSPc, blocked its binding/interaction with intestinal epithelial receptors and impeded larval invasion of intestinal epithelium, thereby reduced infection stress-induced goblet cell proliferation [58]. Furthermore, rTsSPc immunization upregulated the expression of intestinal TJs (E-cad, Occludin and Claudin-1), enhanced intestinal epithelial structural integrity, reduced the reliance on stress-induced mucin secretion, and restored mucus layer homeostasis.

Conventionally, goblet cell hyperplasia is a compensatory and reparative response of the host to intestinal barrier disruption and inflammatory stimulation. It represents not only a component of immune response but also a pathophysiological process [59]. In the infected PBS group, *T. spiralis* infection and sustained stimulation of the worms-secreted TsSPc led to intestinal injury and inflammation. To counteract this damage, host initiated the marked inflammatory cell infiltration, excessive goblet cell hyperplasia, and plentiful of mucus secretion. The increased number of goblet cells observed in infected PBS group is really a state of intestinal pathological stress and hyper-responsiveness. The core function of rTsSPc vaccine is primarily to elicit abundant antibodies capable of specifically neutralizing the TsSPc protease. While challenge infection, these antibodies rapidly bind to worms-derived TsSPc in intestinal mucosal surface, preventing TsSPc from contacting and damaging intestinal epithelium. rTsSPc immunization significantly upregulates the levels of anti-inflammatory cytokines (IL-10 and TGF-β). These cytokines exert potent immunomodulatory functions and suppress excessive inflammatory responses and tissue injury. They might directly or indirectly restrict excessive goblet cell hyperplasia and abnormal mucin secretion resulted from *T. spiralis* infection. Such a regulatory or anti-inflammatory immunological microenvironment aims to eliminate the pathogens at minimal cost while maintaining tissue homeostasis. Under these conditions, the intestine does not undergo severe damage or inflammatory reaction, thus obviating the need to initiate the emergent repair program of massive goblet cell proliferation [60]. In this study, obvious reduction of goblet cells in rTsSPc+infection group serves as a direct evidence that intestinal pathological injury has been successfully prevented or markedly alleviated, and constitutes a robust demonstration of the vaccine’s protective efficacy.

Macrophages are the core effector cells of host immune system, exhibit high plasticity and heterogeneity, and can polarize into distinct functional phenotypes in response to micro-environmental cues. Classically activated (M1) macrophages are primarily induced by stimuli such as IFN-γ and lipopolysaccharide (LPS), highly expressed pro-inflammatory molecules (iNOS, TNF-α and IL-6) mediate pathogen killing, inflammatory responses, and adaptive immune activation. Alternatively activated (M2) macrophages are induced by cytokines such as IL-4, IL-13, IL-10 and TGF-β, highly expressed markers Arg-1, CD206 and chitinase-like protein (Ym1), and exert anti-inflammatory, tissue repair and immune regulatory functions [61]. Peritoneal macrophages from rTsSPc-immunized mice exhibited mixed M1/M2 polarization and shifted toward M2 dominance after infection. This mixed polarization may be attributed to multiple antigenic epitopes within rTsSPc that might simultaneously activate distinct immune cell subsets and induce heterogeneous cytokine profiles (e.g., concurrent Th1/Th2 cytokine production), providing the molecular basis for mixed M1/M2 phenotype [62]. Additionally, local micro-environmental heterogeneity may contribute to the spatial variation in macrophage polarization [63]. The mixed M1/M2 phenotype confers significant biological advantages: M1 macrophages provide potent anti-infective activity via the NO and reactive oxygen species (ROS) production, while activating adaptive immunity through antigen presentation; M2 macrophages mediate anti-inflammatory effects and tissue repair, reducing immunopathological damage and maintaining immune homeostasis [64]. This balance is critical for inducing effective protective immunity while avoiding excessive inflammatory injury post-vaccination. The polarization shift from mixed M1/M2 post immunization to M2 dominance post-infection represents a key mechanism of rTsSPc-induced immunity against *T. spiralis* infection: the mixed phenotype post immunization balances immune activation and inflammatory homeostasis to support adaptive immune priming, while M2 dominance post-infection prioritizes parasite clearance and tissue repair, effectively reduces parasite burden and alleviates pathological damage [65,66]. During *T. spiralis* infection, dynamic changes of cytokine network drive M2 macrophage polarization: the host predominantly produces Th2 and regulatory cytokines, forms a microenvironment conducive to M2 polarization, with IL-4 and IL-13 serving as key inductive factors. This regulatory process not only highlights the central role of macrophages in anti-*T. spiralis* immunity but also provides novel insights for vaccine design—optimizing M1/M2 switching efficiency may further enhance vaccine protective efficacy, supporting TsSPc as a promising anti-*T. spiralis* vaccine candidate.

ADCC assay confirmed that rTsSPc immune serum mediated 81.68% cytotoxicity of peritoneal macrophages killing NBLs, which was significantly higher than the PBS group. When various groups of macrophages were co-cultured with the NBL and anti-rTsSPc serum, the macrophages’ cytotoxicity of the rTsSPc group was also obviously higher than the PBS control group. This synergistic effect between specific antibodies and macrophages constitutes a potent effector system for NBL elimination and prevention of the larval penetration and settlement in skeletal muscles [17,67]. Another critical protective mechanism of rTsSPc immunization might be due to the blockade of interactions between parasite-derived TsSPc and host intestinal epithelial receptors (RACK1, CK8 and PGAM5), and the activation of the corresponding signal pathways [32,68,69]. In this study, IFA results showed that rTsSPc immunization abrogated and decreased TsSPc-specific green fluorescence in intestinal epithelium and significantly reduced the co-localization signal between TsSPc and gut epithelial RACK1 receptors. qPCR and Western blotting results showed that expression levels of RACK1 and MAPK/ERK1/2 pathway proteins in the rTsSPc group after infection were distinctly lower than the PBS group, suggested that anti-rTsSPc antibodies produced by rTsSPc immunization might neutralize the parasite-derived TsSPc and block its binding to RACK1 receptor, subsequently prevent the activation of MAPK/ERK1/2 pathway and damage of gut epithelial integrity, and finally impede the parasite invasion [35,70]. The finding also indicated that the binding of TsSPc to RACK1 increased RACK1 expression. When worms-derived TsSPc was neutralized by anti-rTsSPc antibodies, its binding to RACK1 was blocked, the TsSPc-elevated RACK1 expression level in rTsSPc+infection group was declined, and naturally returned to the basal level of uninfected PBS group. Therefore, anti-rTsSPc antibodies not only prevented the binding of TsSPc to RACK1, but also caused a change in the expression level of RACK1.

Previous studies also showed that at the early stage of infection, the AW number in intestinal tract of male mice is typically significantly higher than the female mice. Additionally, female mice expel intestinal AW more rapidly, leading to a shorter AW survival duration in female hosts [71]. Estrogen, particularly 17β-estradiol (E2), is widely recognized as a key molecule conferring stronger resistance in females. It enhances B cell antibody production, promotes Th2 cell differentiation, strengthens the macrophage’s phagocytic function, and upregulates the expression of several genes associated with anti-helminth immunity. These immunostimulatory effects jointly contribute to the immune advantage of female hosts. Testosterone and other androgens are usually considered to exert immunosuppressive effects; they inhibit lymphocyte proliferation, induce a Th1-skewed immune response, suppress Th2 cytokine production, and potentially reduce antibody production levels. This inherent immunosuppressive tendency renders male hosts relatively weaker in defensive capacity against various pathogen infections [72–73].

Cell-mediated immunity is central to anti-helminth immunity, and the primary immune modality against intestinal helminths is the Th2-type immune response. In contrast, the Th1-type immune response, which primarily targets intracellular pathogens, is often regarded as non-protective in anti-helminth immunity. Gender dimorphism represents one of the mechanisms underlying the differential regulation of Th1/Th2 responses. Studies revealed that estrogen tends to promote Th2-type immune responses, whereas androgens suppress Th2 responses or enhance Th1 responses [74–75]. Gender dimorphism is a well-recognized critical factor in immunological research. As evidenced by the aforementioned studies, female individuals tend to mount a more robust Th2-type humoral immune response under hormonal regulation, which exacerbates the susceptibility disadvantage of males relative to females. In this study, only female BALB/c mice were used for all immunization and challenge experiments. Therefore, the mixed-gender animals should be used in further confirmatory experiment.

Despite the promising results, this study still has several limitations that need to be addressed in future research. The rTsSPc-binding receptors in macrophages and pathway in M1/M2 polarization were not identified and characterized in the present study. The protective efficacy of TsSPc vaccination was verified only in a murine model, while pork is the primary source of human *Trichinella* infection. Hence, the TsSPc immune protection needs to be further validated in a porcine model [4,25,76]. *T. spiralis* has a complex life cycle with stage-specific antigens and TsSPc is a single antigen molecule; therefore, multivalent vaccines consisting of TsSPc with other protective antigens (e.g., C-type lectin, galectin, elastase, etc.) may have higher immune protection [77]. Moreover, the immunization route in this study was subcutaneous injection, while *T. spiralis* infection is acquired by oral ingestion of infected animal meat. Therefore, oral immunization of multivalent vaccines might theoretically elicit stronger mucosal immunity and is more suitable for vaccinating domestic pigs. Additionally, the optimization of immunization routes and adjuvants also deserves further exploration [5].

In conclusion, immunization of mice with rTsSPc induced robust protective immunity against *T. spiralis* infection. This immune protection was obtained through multiple synergistic mechanisms: eliciting strong systemic Th2-biased Th1/Th2 humoral immunity as well as cellular immunity, and gut local mucosal sIgA responses; enhancing intestinal epithelial integrity and barrier function, regulating gut inflammatory responses to inhibit larval invasion; modulating peritoneal macrophage polarization to enhance the macrophages’ ability of ADCC killing newborn larvae. Additionally, anti-rTsSPc antibodies produced by rTsSPc vaccination might neutralize the parasite-derived TsSPc and block its binding to gut RACK1 receptors, subsequently intercept the activation of MAPK/ERK1/2 pathway and destruction of gut epithelial integrity, and finally impede the larval invasion. Collectively, these findings indicated that TsSPc might be a promising novel candidate target for anti-*T. spiralis* vaccine, and provides a new insight of prevention and control strategies of *Trichinella* infection.

## Supporting information

S1 Table Raw data of Figs 1–9 in this study. (XLSX)
